# Editorial: Advances in Density Functional Theory and Beyond for Computational Chemistry

**DOI:** 10.3389/fchem.2021.705762

**Published:** 2021-07-12

**Authors:** Wei Hu, Mohan Chen

**Affiliations:** ^1^ Hefei National Laboratory for Physical Sciences at the Microscale, University of Science and Technology of China, Hefei, China; ^2^ Computational Research Division, Lawrence Berkeley National Laboratory, Berkeley, CA, United States; ^3^ HEDPS, CAPT, College of Engineering, Peking University, Beijing, China

**Keywords:** density functional theory, low-scaling algorithms, machine learning, excited-state, high-performance computing

The rapid development of modern computational chemistry has led to a growing need to understand the microscopic mechanisms determining the properties of molecular and solid materials at an atomic level. The interactions between atoms and electrons are governed by the laws of quantum mechanics; hence, accurate and efficient computational methods for solving the quantum-mechanical equations are needed. The Kohn-Sham density functional theory (DFT) Hohenberg and Kohn (1964), Kohn and Sham (1965) marks a decisive breakthrough in these efforts, and in the past few decades DFT has made an unparalleled impact on a variety of interesting and challenging problems in computational chemistry. The real forte of DFT is its favourable price and performance ratio as compared with electron-correlated wave-function-based methods, such as the Møller–Plesset perturbation theory Binkley and Pople (1975) or coupled cluster theory Čížek (1966). Thus, large-scale molecular and solid systems can be studied by DFT with sufficient accuracy, thereby expanding the predictive power inherent in electronic structure theory. As a result, DFT is now by far the most widely used electronic structure method. Although 50 years have passed since the formulation of the Kohn-Sham DFT, many open questions remain, including the mathematical issues in solving the Kohn-Sham equations, the developments of more accurate and efficient density functionals, and applying the DFT calculations to solve more scientific problems. This research topic focuses on covering recent advances within the framework of DFT.

Computational chemistry methods have become increasingly important in recent years, as manifested by their rapidly extending applications in a large number of diverse fields, such as computations of molecular structures and properties, the design of pharmaceutical drugs and novel materials, etc. In part as a result of this general trend, the size of the systems which can be computationally studied has also increased, generating even further needs for large-scale applications. This is because larger molecular systems show interesting phenomena and have important implications in modern biochemistry, biotechnology, and nanotechnology. Thus, it is of great importance to apply and further develop computational methods which provide physically sound models for large molecules at a reasonable computational cost. A representative approach is the linear scaling technique Goedecker (1999), which owns a computational cost that scales linearly O(N) with the size of the system. The linear-scaling DFT is an area of active research in computational chemistry, with the performances improve steadily over the years, especially on parallel high-performance machines. Historically, linear-scaling implementations were restricted to basic ground state energy and electron density calculations, but this has also improved in recent years with geometry optimizations and molecular dynamics (MD) becoming available. Moreover, recent developments of machine learning algorithms enable the large-scale MD simulations with *ab initio* accuracy, and has been applied to a variety of applications Jia et al. (2020). This research topic aims to report the state-of-the-art computational methods in several of the important questions related to the family of linear scaling methods.

A deep understanding of the excitations in molecules and solids are of fundamentally importance in many technological applications. There is already a rich set of theoretical and simulation methods for excited-state calculations, such as the GW plus Bethe-Salpeter equation Hedin (1965), time-dependent density functional theory (TDDFT) Runge and Gross (1984) and many-body coupled cluster (CC) theory Čížek (1966). Unfortunately, these post-Hartree-Fock and excited state methods for electronic excitations are all subject to computational bottlenecks, which are far more severe than those affecting the standard calculations of the ground-state energy, not only because of the system size, but also because the large number of excited states that need to be considered. A major difficulty for treating excited complex systems arises from the different nature of the various competing excited electronic states. For example, the localized neutral and delocalized charge transfer excitons, as a result of the relatively large length scale. Therefore, this research topic also aims to cover developments of novel electronic structure algorithms and scalable computational methods for excited states of complex systems.

The past several decades have witnessed tremendous strides in the capabilities of computational chemistry simulations, driven in large part by the extensive parallelism offered by powerful computer clusters and scalable programming methods on high performance computing (HPC) Hu et al. (2021), Kowalski et al. (2021). However, such massively parallel simulations increasingly require more advanced algorithms to achieve satisfactory performance across the vastly diverse ecosystem of modern heterogeneous computer systems. The design of efficient parallel codes proves to be difficult: the diversity of involved data structures and algorithms, as well as the frequently occurring inherent sequential control propose enormous challenges to efficiently use of a large number of processors. This research topic also focuses on the developments of more effective computational methods by use of high performance parallel computing.

This editorial sums up the contents of our Research Topic “Advances in Density Functional Theory and Beyond for Computational Chemistry” and a total of nine original research contributions have been included in this article collection, involving linear-scaling density functional theory, multiple scattering theory, ab initio molecular dynamics, deep potential model, hybrid and double-hybrid functional theory, second-order Møller–Plesset perturbation theory, coupled cluster theory and high performance computing.

Linear-scaling DFT Goedecker (1999) is an efficient method to yield the structural and electronic properties of molecules, semiconductors, and insulators to avoid the high cubic-scaling cost in conventional DFT calculations. Luo et al. described an efficient parallel implementation of linear-scaling density matrix trace correcting purification algorithm Niklasson (2002) to solve the Kohn–Sham equations with numerical atomic orbitals in the HONPAS Qin et al. (2015) package. The authors have performed large-scale DFT calculations on boron nitrogen nanotubes containing tens of thousands of atoms, which can scale up to hundreds of processing cores on modern heterogeneous supercomputers.

The Korringa–Kohn–Rostoker Green’s function method Korringa (1947), Kohn and Rostoker (1954), also known as multiple scattering theory (MST) Lloyd and Smith (1972), provides equivalent information as solving the Kohn-Sham equation by employing the single-particle Green’s function Economou (2006). Cao et al. investigated a reduced scaling full-potential DFT method based on the multiple scattering theory code MuST Rusanu et al. (2011). A significant advantage of the MST method is the reduced scaling in the calculations of metallic systems. The MST method shows the potential to simulate more complicated materials on massively parallel supercomputers and provides a reliable and accessible way to large-scale first-principle simulations of metals and alloys.

AIMD (*ab initio* molecular dynamics) has been extensively employed to explore the dynamical information of electronic systems. However, it remains extremely challenging to reliably predict electronic properties of systems with a radical nature using first-principles DFT calculations due to the presence of the static correlation. To address this challenge, Li and Chai proposed TAO-DFT (thermally-assisted-occupation density functional theory) with AIMD to explore the dynamical properties of nanosystems with a radical nature at finite temperatures. A variety properties of *n*-acenes (*n* = 2–8) at 300 K are presented including the instantaneous/average radical nature and infrared spectra of *n*-acenes containing *n* linearly fused benzene rings (*n* = 2–8).

Predicting crystal structure has been a challenging problem, which requires a reliable energy calculation engine and an efficient global search algorithm. Machine learning based inter-atomic potential energy surface models such as the deep potentials Jia et al. (2020) owns the DFT accuracy and the speed of empirical force fields and can be used as an energy calculator. Wang et al. employed the deep potential model to predict the intermetallic compound of the aluminum–magnesium system, and found six meta-stable phases with negative or nearly zero formation energy. The authors proposed a relatively robust structure screening criterion that selects potentially stable structures from the Deep Potential-based convex hull and performs DFT refinement. By using this criterion, the computational cost needed to construct the convex hull with ab initio accuracy can be dramatically reduced.

Accurate prediction of quasiparticle and excitation energies has been very challenging for ground-state density functional methods since the commonly adopted density functional approximations suffer from the delocalization error. Yang et al. propsed a new method which presumed a quantitative correspondence between the quasiparticle energies and the generalized Kohn–Sham orbital energies. Furthermore, the authors employed a previously developed global scaling correction approach to achieve substantially improved prediction of molecular quasiparticle and excitation energies.

Interpretation of spectroscopic experiments is challenging because the results are affected by the interplay of stereo-electronic, dynamic and environmental effects. The work of Barone et al. showed that the last-generation hybrid and double-hybrid functionals Biczysko et al. (2010), which are described by partially augmented double- and triple-zeta basis sets, provided unprecedented accuracy for medium-size semi-rigid molecules under the framework of the second order vibrational perturbation theory.

The second-order Møller–Plesset perturbation theory (MP2) Binkley and Pople (1975) is a post-Hartree–Fock approach to taking the electron correlation effect into account. Despite its simple form, the MP2 method captures around 90% of the correlation energy Bartlett and Stanton (1994), but the $O\left(N^{5}\right)$ computational scaling of the original (canonical) MP2 method has limited the application of the MP2 method in large systems. Shang and Yang implemented the canonical and Laplace-transformed algorithms to calculate the MP2 perturbation theory for the total energy and the band gap of periodic systems under periodic boundary conditions in HONPAS Qin et al. (2015) code with numerical atomic orbitals. The MP2 correction energy and band gaps presented in the work are in excellent agreement with the results of the canonical MP2 formulation. Moreover, the authors studied the binding-energy curves for the two stacked transpolyacetylene chains and demonstrated that the new method well describe the correlation energy and the long-range van der Waals interactions.

The coupled cluster (CC) theory Čížek (1966) has become one of the most accurate ab initio methods to yield the electronic structure information. Yang et al. presented a Newton Krylov method Knoll and Keyes (2004) for solving the coupled cluster equation. This new method used a Krylov subspace iterative method, such as the Generalized Minimum Residual (GMRES) method Saad and Schultz (1986), to compute the Newton correction to the approximate coupled cluster amplitude. Numerical results demonstrate the effectiveness and robustness of the Newton Krylov method not only for standard CCSD calculations but also for tailed CCSD calculations where the information for external correction is obtained from a density matrix renormalization group (DMRG) calculation Schollwöck (2005).

Williams-Young et al. proposed a three-level parallelism scheme for the distributed numerical integration of the exchange-correlation potential in the Gaussian basis set discretization of the Kohn–Sham equations on large computing clusters consisting of multiple graphics processing units (GPU) per compute node. They demonstrated that the performance and scalability of the implementation of the purposed method in the NWChemEx Kowalski et al. (2021) software package by comparing to the existing scalable CPU exchange-correlation integration in NWChem. The results show that the speedups are between 10× and 100× as compared to the analogous CPU implementation in NWChem.

The above article collection demonstrates that the DFT methods have broad impacts on a variety of subjects in computational chemistry and related disciplines. In conjunction with high-performance computation and machine-learning techniques, the DFT framework undergoes another round of fast developments. It can be expected that more accurate DFT approaches with more efficient algorithms will be available in the near future.
